# A Novel Prognostic Signature Based on Ferroptosis-Related Genes Predicts the Prognosis of Patients With Advanced Bladder Urothelial Carcinoma

**DOI:** 10.3389/fonc.2021.726486

**Published:** 2021-12-13

**Authors:** Xiaoqi Li, Junting Huang, Ji Chen, Yating Zhan, Rongrong Zhang, Enze Lu, Chunxue Li, Yuxiao Zhang, Yajing Wang, Yeping Li, Jianjian Zheng, Wujun Geng

**Affiliations:** ^1^ Key Laboratory of Diagnosis and Treatment of Severe Hepato-Pancreatic Diseases of Zhejiang Province, The First Affiliated Hospital of Wenzhou Medical University, Wenzhou, China; ^2^ Department of Urology, The First Affiliated Hospital of Wenzhou Medical University, Wenzhou, China; ^3^ Department of Anesthesiology, The First Affiliated Hospital of Wenzhou Medical University, Wenzhou, China; ^4^ Wenzhou Key Laboratory of Perioperative Medicine, The First Affiliated Hospital of Wenzhou Medical University, Wenzhou, China

**Keywords:** ferroptosis, prognostic signature, bladder urothelial carcinoma, immune status, function analyses

## Abstract

Bladder Urothelial Carcinoma (BLCA) is the major subtype of bladder cancer, and the prognosis prediction of BLCA is difficult. Ferroptosis is a newly discovered iron-dependent cell death pathway. However, the clinical value of ferroptosis-related genes (FRGs) on the prediction of BLCA prognosis is still uncertain. In this study, we aimed to construct a novel prognostic signature to improve the prognosis prediction of advanced BLCA based on FRGs. In the TCGA cohort, we identified 23 differentially expressed genes (DEGs) associated with overall survival (OS) *via* univariate Cox analysis (all *P* < 0.05). 8 optimal DEGs were finally screened to generate the prognostic risk signature through LASSO regression analysis. Patients were divided into two risk groups based on the median risk score. Survival analyses revealed that the OS rate in the high-risk group was significantly lower than that in the low-risk group. Moreover, the risk score was determined as an independent predictor of OS by the multivariate Cox regression analysis (Hazard ratio > 1, 95% CI = 1.724-2.943, *P* < 0.05). Many potential ferroptosis-related pathways were identified in the enrichment analysis in BLCA. With the aid of an external FAHWMU cohort (n = 180), the clinical predication value of the signature was further verified. In conclusion, the prognosis of advanced BLCA could be accurately predicted by this novel FRG-signature.

## Introduction

Bladder cancer (BC), ranking 9^th^ in the incidence of malignant tumors, is a malignant tumor of the bladder mucosa with poor prognosis and high recurrence (1, 2). Usually, BC could be divided into muscle invasive bladder cancer (MIBC) and non-muscle invasive bladder cancer (NMIBC). In comparison with patients with NMIBC, patients with MIBC have a poor prognosis. Bladder Urothelial Carcinoma (BLCA), caused by cigarette smoking and occupational exposure, is the main BC subtype, accounting for 90–95% of cases (3, 4). However, clinical biomarkers that could accurately predict the prognosis of BLCA are still lacking. Most BLCA patients are diagnosed at the late stages, leading to the worse prognosis (4). Therefore, the need of a better predictive signature for the prognosis prediction of BCLA, especially for MIBC, is urgent.

Ferroptosis, results from the accumulation iron-dependent lipid peroxide (LPO), is a new discovered pathway of non-apoptotic cell death (5). Ferroptosis is mainly caused by iron-dependent oxidative damage (6). It has been reported to be involved in the vital biological functions, including a sequence of complex biochemical reactions, gene expression, and signal transduction events (7, 8). Ferroptosis-related genes (FRGs) has been demonstrated to be associated with the prognosis of various human cancers (9–12). However, the clinical value of FRGs in the prognosis of BLCA still remains unknown.

In this study, we constructed a novel prognostic signature to improve the prognosis prediction of BLCA. Due to the reason that most patients with BLCA in TCGA database are with advanced-stage disease, we focused on the clinical value of FRGs in patients with MIBC. With the aid of an extra cohort obtained from the First Affiliated Hospital of Wenzhou Medical University (FAHWMU), the accuracy of this prognostic signature was verified.

## Materials and Methods

### Data Collection

In this study, mRNA expression files [fragment per thousand base pairs per kilobase fragment normalization (FPKM) normalized] and clinical features from 406 BLCA patients were obtained from the TCGA database (https://portal.gdc.cancer. Government/Repository). Using the R package “limma”, gene expression profiles were standardized. Data from TCGA are publicly available and use standardized read count values. As this study follows the TCGA data access strategy and release guidelines, it does not require approval by the local ethics committee. The 259 FRGs were selected from the previous studies (13, 14). As an extra cohort data, the FAHWMU cohort (n = 180) was obtained from the First Affiliated Hospital of Wenzhou Medical University (Wenzhou, China). BLCA samples in the FAHWMU cohort were collected from 2012 to 2020, and OS time was used as the main survival time indicator. The collection of this cohort was reviewed and approved by the human research ethics committee of the First Affiliated Hospital of Wenzhou Medical University. The patients/participants provided their written informed consent to participate in this study.

### Generation and Validation of the Signature

In the TCGA cohort, differentially expressed genes (DEGs) between BLCA samples and adjacent nontumorous samples were identified using “limma” with a false discovery rate (FDR) < 0.05. Prognosis related DEGs were screened using univariate Cox analysis of overall survival (OS) (*P* < 0.05), *P*-values were adjusted using Benjamini & Hochberg (BH) correction. The STRING database (version 11.0) generated a protein-to-protein interaction (PPI) network for the interactions among prognostic DEGs (15). The prognostic signature was constructed using the combination of LASSO-penalized Cox regression and multivariate Cox regression (16, 17). The LASSO algorithm was used to select and compress variables. In the regression, the standardized expression matrix of the prognosis related DEGs constituted the independent variable, while the OS and patient status in the TCGA cohort constituted the response variables. Standard ten-fold cross-validation determined the model penalty parameter (λ). The standardized expression level and regression coefficients for each gene were used to calculate patient risk, where the score = sum (each gene’s expression × corresponding coefficient). In the TCGA cohort, as determined by the median risk score, all patients were divided into the high-risk and low-risk groups. By using the “prcomp” function from the R package “stats”, PCA was performed after the gene expression signature was obtained. t-SNE, produced by the R package “Rtsne”, explored group distribution. Time-dependent receiver operating characteristic (ROC) curve analysis was used to assess the performance of gene signatures. The Kaplan-Meier (K-M) survival analysis was performed using the log rank test.

### Enrichment Analysis

DEGs (|log2FC|≥1, FDR < 0.05) were identified using the R package “clusterProfiler” based on the risk score. We used the BH method to adjust P values based on Gene Ontology (GO) function enrichment analysis and Kyoto Encyclopedia of Genes and Genomes (KEGG) pathways enrichment analysis. The single-sample gene set enrichment analysis (ssGSEA) was used to calculate the activity of 13 immune-related pathways and the infiltration analysis data of 16 immune cells (18). The gene set of the 13 immune-related pathways and the 16 immune cells is shown in **Table S2**.

### Statistical Analysis

Student’s t-test was performed to compare gene expression between BLCA and controls. The Mann-Whitney test was used to compare ssGSEA scores between high- and low-risk groups. *P* values < 0.05 were considered statistically significant. R software (Version 3.5.3) was used for all statistical analyses.

## Results

### Identification of 23 OS-Related Differently Expressed Ferroptosis-Related Genes

**Figure 1:**
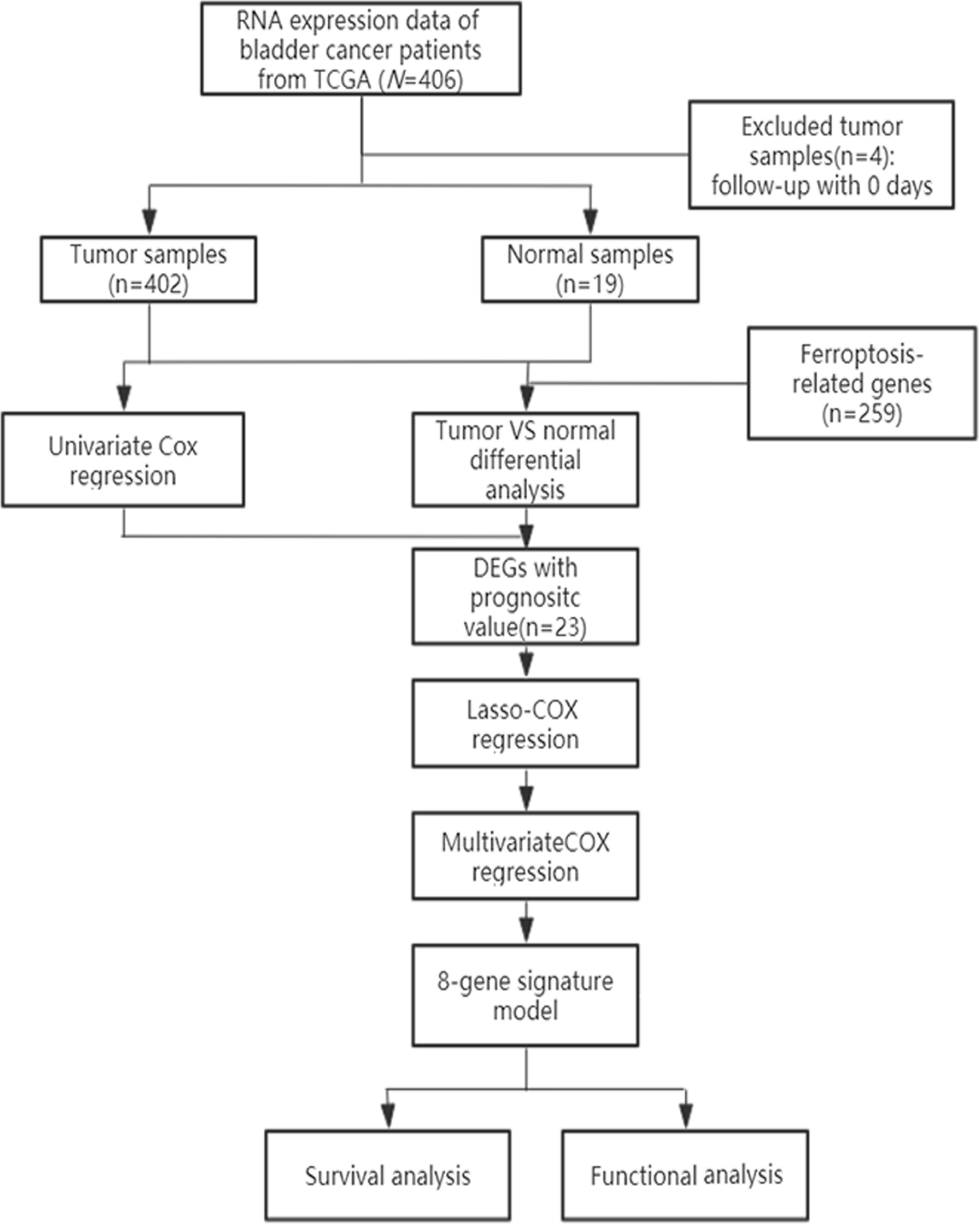
Flowchart of data collection and analysis.

A schematic representation of data collection and analysis was shown in **Figure 1**. We obtained data for BLCA patients from the TCGA databases. Clinical characteristics were summarized in **Table 1**.

**Table 1:** The clinical features of TCGA cohort and the FAHWMU cohort.

	TCGA cohort	FAHWMU cohort
**No.of patients**	406	180
**gender**		
female	106 (26.1%)	47 (26.1%)
male	300 (73.9%)	133 (73.9%)
**Age**		
>65	245 (60.3%)	107 (59.4%)
≤65	161 (39.7%)	73 (40.5%)
**status**		
alive	227 (55.9%)	93 (51.7%)
dead	179 (44.1%)	87 (48.3%)
**stage**		
Stage I	2 (0.5%)	21 (11.7%)
Stage II	129 (31.8%)	56 (31.1%)
Stage III	140 (34.5%)	44 (24.4%)
Stage IV	133 (32.8%)	57 (31.7%)
unknown	2 (0.5%)	2 (1.1%)

**Figure 2:**
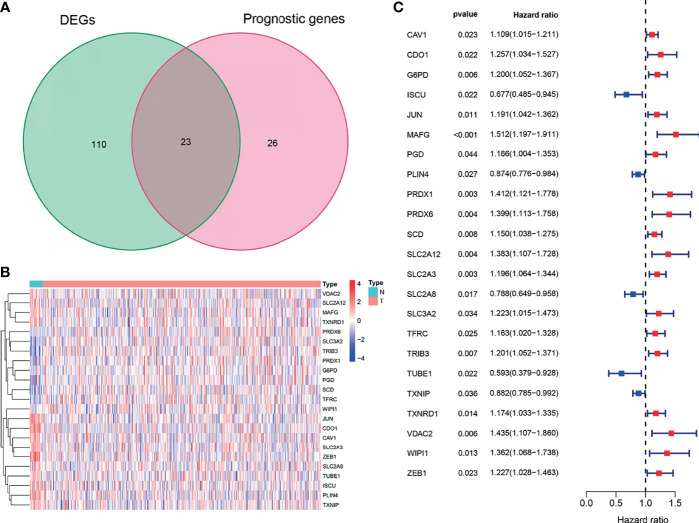
Identification of OS-related DEFRGs in the TCGA cohort. **(A)** Venn plot showing common genes from differential analysis and univariate Cox analysis. **(B)** Heat map of OS-related DEFRGs. **(C)** Forest plots showing OS-related DEFRGs (*P* < 0.05).

**Figure 3:**
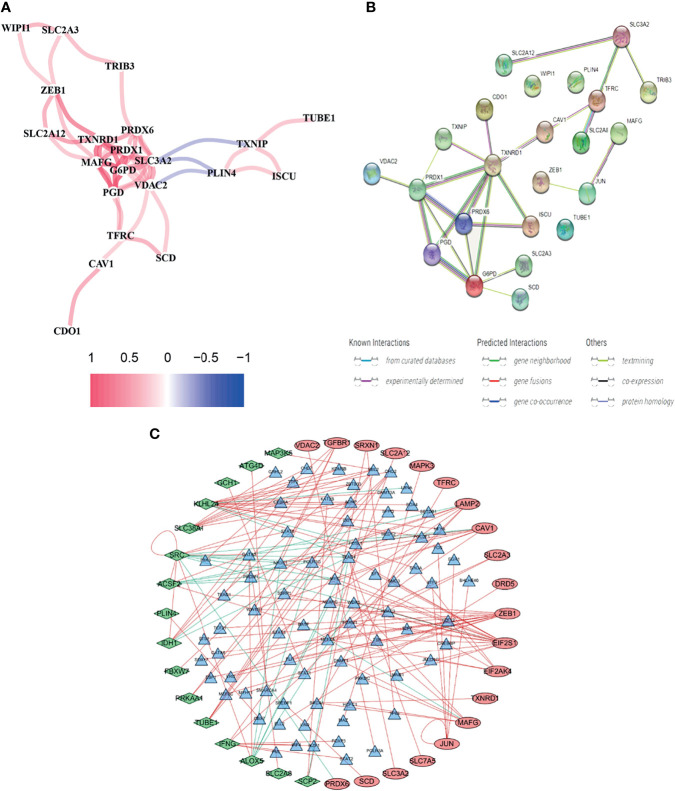
Comprehensive networks of 23 OS-related DEFRGs. **(A)** PPI network **(B)** Correlation network of 23 OS-related DEFRGs. **(C)** Regulatory network of TFs and DEFRGs (green nodes: DEFRGs with low risk; red nodes: DEFRGs with high risk, only nodes with correlation coefficient > 0.4 and *P* < 0.05 were selected).

Using BLCA samples from the TCGA cohort, differential expression between BLCA samples and adjacent nontumorous samples was observed in most FRGs (133/259, 51.4%); 23 were related to OS *via* univariate Cox regression (all *P* < 0.05, **Figures 2A**–**C**). The gene interaction network indicated that PRDX6, TXNRD1, PRDX6, and G6PD were the hub genes (**Figure 3A**). The correlations among genes were shown in **Figure 3B**. It was found that there were significant relationships between 193/318 TFs and DEGs (*P* < 0.05). A TF-based regulatory network was shown in **Figure 3C**.

### Generation of the 8 Ferroptosis-Related Prognostic Gene Signature

**Figure 4:**
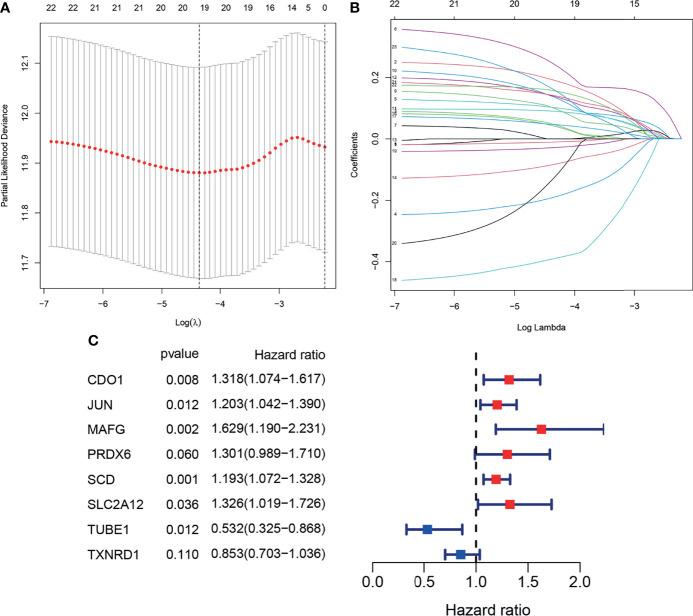
Construction of the prognostic model. **(A, B)**. Lasso-Cox analysis of 23 OS-related DEFRGs. **(C)** Forest plot showing the eight genes in the prognostic risk model.

To prevent over fitting of the model, Lasso-Cox regression was used to screen the identified 21 genes (**Figures 4A, B**). Then, multivariate Cox regression analysis was used to determine the optimal regulatory genes and 8 FRGs were identified to generate the prognostic signature (**Figure 4C**). The risk scores of BLCA patients were calculated using mRNA levels and the estimated regression coefficients. The resulting formula was used:


$$\begin{array}{c} \text{Training cohort risk score}=(0.2758\times \text{expression of CDO}1)+(0.1851\times \text{expression of JUN})\\ +(0.4881\times \text{expression of MAFG})+(0.2629\times \text{expression of PRDX}1)\\ +(0.1766\times \text{expression of SCD})+(0.2825\times \text{expression of SLC}2A12)\\ +(-0.6320\times \text{expression of TUBE}1)+(-0.1586\times \text{expression of TXNRD}1). \end{array}$$

**Figure 5:**
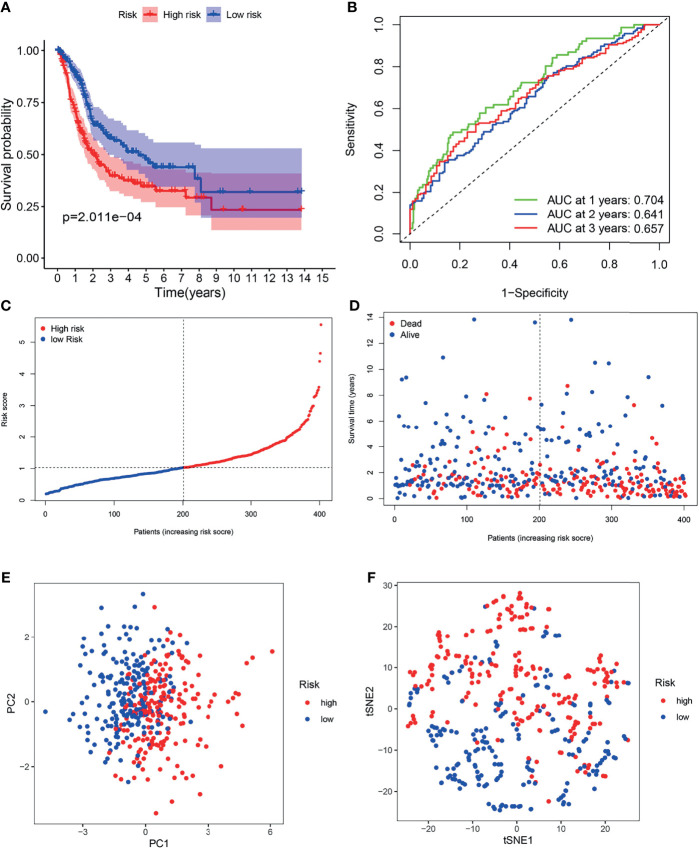
Survival analysis of the TCGA cohort prognostic signature. **(A)** Kaplan-Meier survival curve. **(B)** time-dependent ROC curve. **(C)** The distribution and median value of the risk scores. **(D)** OS-related scatter plot. **(E)** PCA plot. **(F)** t-SNE plot.

Patients in the TCGA cohort were partitioned into high-risk (n = 203) and low-risk groups (n = 203) according to the median risk score (**Figure 5C**). K-M survival curves indicated that the OS of the low-risk group was higher than that of the high-risk group (**Figure 5A**, *P* < 0.05). Time-dependent ROC curves indicated that area under the curve (AUC) values were 0.704, 0.641, and 0.657 at 1^st^ years, 2^nd^ years and 3^rd^ years (**Figure 5B**). As shown in **Figure 5D**, worse OS was found in high-risk patients, consistent with the results of the K-M curve. PCA and t-SNE analysis indicated two directions in the patient distribution of the two risk groups (**Figures 5E, F**).

### Validation of the Prognostic Value of Gene Signature in the FAHWMU Cohort

**Figure 6:**
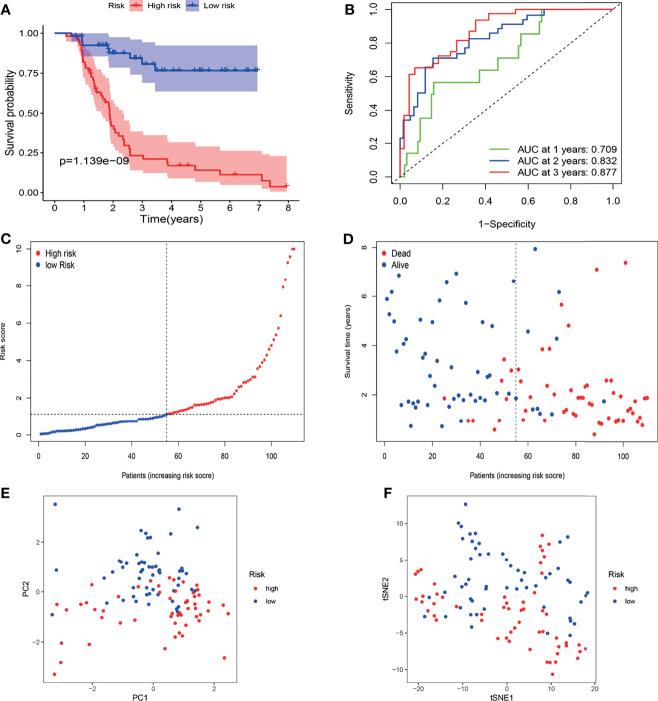
Validation of gene signature in patients with MIBC in the FAHWMU cohort (n = 110). **(A)** Kaplan-Meier survival curve. **(B)** time-dependent ROC curve. **(C)** The distribution and median value of the risk scores. **(D)** OS-related scatter plot. **(E)** PCA plot. **(F)** t-SNE plot.

The FAHWMU cohort was used to verify prognostic signature. Considering that most patients with BLCA in TCGA database are at the advanced-stage, only patients with MIBC in the FAHWMU cohort (n = 110) were used for the next analysis. The risk score was calculated based on the expressions of eight risk genes: CDO1, JUN, MAFG, PRDX6, SCD, SLC2A12, TUBE1 and TXNRD1. According to the calculated median risk score of the FAHWMU cohort, all patients were divided into high-risk (n=55) or low-risk (n=55) groups (**Figures 6C, D**). Similarly, the AUC in 1^st^ years, 2^nd^ years and 3^rd^ years in the FAHWMU cohort was 0.709, 0.832, and 0.877 (**Figure 6B**). K-M survival curve indicated that compared to low-risk patients, the OS of high-risk patients was worse (**Figure 6A**, *P* < 0.05). The results of PCA and t-SNE analysis in the FAHWMU cohort were similar to the results of the TCGA cohort (**Figures 6E, F**). Taken together, our results preliminarily suggest the prognosis prediction of our signature in BLCA.

### The Risk Score Was Identified as an Independent Prognostic Predictor

**Figure 7:**
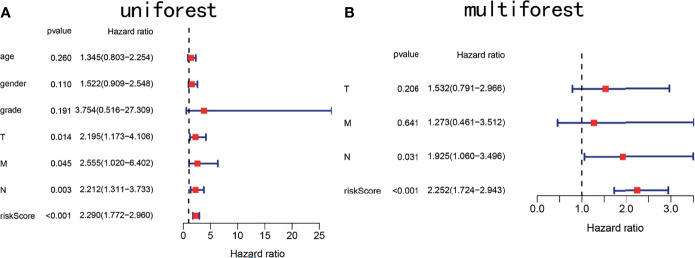
Forest plots showing key clinical factors under the signature. **(A)** Univariate Cox regression analysis of the TCGA cohort. **(B)** Multivariate Cox regression analysis of the TCGA cohort.

To determine whether the risk score is an independent prognostic marker, univariate and multivariate Cox regression analyses were performed. Univariate Cox regression indicated that the risk score of TCGA cohort was closely related to OS (*P* < 0.001, **Figure 7A**). Multivariate Cox regression analysis further confirmed the risk score as an independent prognostic factor in TCGA cohort (*P* < 0.01, **Figure 7B**).

### Function Enrichment Analyses Revealed Potential Molecular Mechanisms

**Figure 8:**
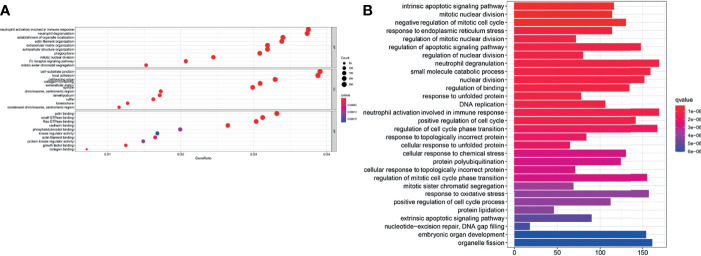
Enrichment analyses of the signature. **(A)** GO enrichment plot of the TCGA cohort. **(B)** KEGG pathway enrichment plot of the TCGA cohort.

GO enrichment and KEGG pathway analysis were performed to reveal ferroptosis-related potential molecular mechanisms. In the TCGA cohort, various neutrophil-related molecular functions and mitotic nuclear division molecular functions were enriched in the GO analysis (*P* < 0.05, **Figure 8A**). KEGG pathway analysis indicated that DNA replication pathways were enriched in the TCGA cohort (*P* < 0.05, **Figure 8B**).

**Figure 9:**
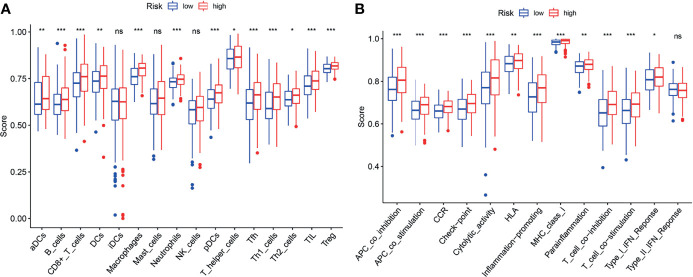
ssGSEA analyses of the signature. **(A, B)** The block diagram showing the scores of 16 immune cells **(A)** and 13 immune-related functions **(B)** of the TCGA cohort. *p < 0.05; **p < 0.01; ***p < 0.001; ns, no significance.

ssGSEA was performed to analyze the relationships between the risk scores and immune-related cells and pathways in TCGA. In the TCGA cohort, there were significant differences between the low-risk and high-risk groups (all adjusted *P* < 0.05, **Figures 9A, B**), with the differences in aDC, Tfh, Inflammation-promoting and MHC class I.

## Discussion

It has been reported that FRGs are involved in the regulation of drug-induced ferroptosis in BLCA (11). Additionally, FRG-related inducers have been found to suppress BC with the help of mTOR inhibitors (19). Recently, ferroptosis participates in the inhibition of cell cycle in BC by obstructing the G0/G1 phase (11). Combined with these, ferroptosis plays a key role in BLCA development. However, the clinical value of FRGs in predicting the OS of BLCA patients was still unknown. In this study, a novel FRG-signature for BLCA was generated. The relationships between FRGs and prognosis of BLCA were explored. Next, the hub FRGs were identified and used to construct this FRG-signature. Our data suggest that this signature could improve the prognosis of advanced BLCA. Notably, the clinical prediction value of our FRG-signature was confirmed in an external FAHWMU cohort.

Herein, 8 FRGs (CDO1, JUN, MAFG, PRDX6, SCD, SLC2A12, TUBE1, TXNRD1) were used to establish the prognostic signature. These FRGs could be summarized as four types: iron metabolism (CDO1), lipid metabolism (JUN, TUBE1), (anti)oxidant metabolism (TXNRD1, SCD, SLC2A12) (20) and energy metabolism (MAFG, PRDX6). CDO1, silenced by promoter methylation in BLCA, induces a reduction in the onset of ferroptosis, leading to the inhibition of BLCA invasion (21). JUN has been reported to reduce lipid peroxidation and inhibit ferroptosis, which contributes to the invasion and metastasis of BC (22). Pitsava et al. found that TUBE1 suppresses BLCA metastasis by promoting ferroptosis through lipid metabolism (23). High expression of SLC2A12 has been found to be associated with glucose metabolism in ferroptosis, which exacerbates the invasion of BLCA (24). PRDX6 could inhibit the high oxidative damage caused by ferroptosis and thus promote the proliferation of BLCA (25). In addition, SCD promotes the proliferation of BLCA *via* fatty acid metabolic pathway in ferroptosis (26). In sum, these 8 FRGs may participate in the key biological processes such as proliferation, invasion and metastasis of BLCA through ferroptosis pathway.

Increasing studies have shown that there is an association between ferroptosis and tumor immunity (27). In this study, it was found that biological functions associated with immunity such as neutrophil activation in immune response, are enriched in the GO analysis. Moreover, it was found that the proportion of macrophages (28, 29) and Treg cells (29, 30) is increased in the high-risk group. Furthermore, several anti-tumor immunity factors, like NK cells, type I IFN response and type II IFN response were decreased in the high-risk group. Recent studies have demonstrated that high proportion of tumor-related macrophages or Treg cells is associated with poor prognosis of BLCA patients. Accordingly, our results also showed that the high-risk patients with the high proportion of macrophages and Treg cells, were associated with poor prognosis, suggesting the existence of the association of ferroptosis and tumor immunity.

The strengths of this study are that an external FAHWMU cohort was used to further verify the clinical value of our FRG-signature in the prognosis prediction of advanced BLCA. Meanwhile, there are several limitations in this study. First, cell biological experiments should be performed to further validate the functions of 8 FRGs in the future. Second, more sample validation is needed for clinical value of our prognostic signature.

## Conclusion

In conclusion, we generate a novel prognostic FRG-signature, which contributes to the improvement in the prognosis prediction of advanced BLCA.

## Data Availability Statement

Publicly available datasets were analyzed in this study. This data can be found here: https://www.ncbi.nlm.nih.gov/geo/https://portal.gdc.cancer.gov/.

## Ethics Statement

The studies involving human participants were reviewed and approved by Human Research Ethics Committee in The First Affiliated Hospital of Wenzhou Medical University. The patients/participants provided their written informed consent to participate in this study. Written informed consent was obtained from the individual(s) for the publication of any potentially identifiable images or data included in this article. The patients/participants provided their written informed consent to participate in this study.

## Author Contributions

XL and WG designed the study and analyzed the data. JH, JC, YTZ, and RZ revised the images. EL, CL, YXZ, and YW performed the literature search and collected data for the manuscript. YL and JZ revised the manuscript. All authors contributed to the article and approved the submitted version.

## Funding

The project was supported by the Zhejiang Provincial Research Center for Cancer Intelligent Diagnosis and Molecular Technology (JBZX-202003).

## Conflict of Interest

The authors declare that the research was conducted in the absence of any commercial or financial relationships that could be construed as a potential conflict of interest.

## Publisher’s Note

All claims expressed in this article are solely those of the authors and do not necessarily represent those of their affiliated organizations, or those of the publisher, the editors and the reviewers. Any product that may be evaluated in this article, or claim that may be made by its manufacturer, is not guaranteed or endorsed by the publisher.

## Abbreviations

BC, Bladder cancer; LASSO, Least absolute shrinkage and selection operator; TCGA, The Cancer Genome Atlas; DEGs, Differentially expressed genes; OS, Overall survival; ROC, Receiver operating characteristic; FDR, False discovery rate; GO, Gene Ontology; KEGG, Kyoto Encyclopedia of Genes and Genomes; ssGSEA, Single-sample gene set enrichment analysis; PCA, Principal component analysis; t-SNE, t-distributed stochastic neighbor embedding; AUC, Area under the curve; HR, Hazard ratio; CI, Confidence interval; APC, Antigen presenting cell; AFP, alpha fetoprotein; aDC, Activated dendritic cell; iDC, Immature dendritic cell; pDC, Plasmacytoid dendritic cell; Tfh, T follicular helper cell; TIL, Tumor Infiltrating Lymphocyte; HLA, Human leukocyte antigen; CCR, Cytokine-cytokine receptor.
